# Roadmap to Dystocia Management—Guiding Obstetric Interventions in Cattle

**DOI:** 10.3390/life15030457

**Published:** 2025-03-13

**Authors:** Nasreddine Larbi Smail, Mounir Adnane, Karen Wagener, Marc Drillich, Aspinas Chapwanya

**Affiliations:** 1 Department of Biomedicine, Institute of Veterinary Sciences, University of Tiaret, Tiaret 14000, Algeria; arbito68@hotmail.fr; 2 Clinical Unit for Herd Health Management, Clinical Centre for Ruminant and Camelid Medicine, Clinical Department for Farm Animals and Food System Science, University of Veterinary Medicine Vienna, 1210 Vienna, Austria; karen.wagener@vetmeduni.ac.at; 3 Unit for Reproduction Medicine and Udder Health, Faculty of Veterinary Medicine, Freie Universität Berlin, 14163 Berlin, Germany; marc.drillich@fu-berlin.de; 4 Department of Clinical Sciences, Ross University School of Veterinary Medicine, Basseterre 00265, Saint Kitts and Nevis; achapwanya@rossvet.edu.kn

**Keywords:** dystocia management, cattle, roadmap strategy, advanced diagnostics, preventive measures, economic impact

## Abstract

Dystocia, or difficult labor, is a common complication during parturition in cattle that poses substantial risks to both dam and fetus. When the incidence is high on a farm level, it is a significant economic burden for dairy and beef enterprises. This review paper presents a comprehensive roadmap strategy to enhance decision-making in the management of dystocia in cows. The strategy encompasses early recognition and assessment, utilization of advanced diagnostic tools, and a range of medical and surgical interventions tailored to specific maternal and fetal causes of dystocia. The roadmap also integrates preventive measures to reduce the incidence of dystocia through genetic selection and optimized nutrition. By addressing the key challenges in dystocia management, such as resource constraints, timely intervention, and the need for continuous education, this strategy aims to improve health outcomes for cows and calves and reduce economic losses. Implementing this structured approach can facilitate better preparedness, efficient resource utilization, and improved overall livestock management, thereby promoting the sustainability and productivity of the cattle industry and addressing animal welfare aspects.

## 1. Introduction

Eutocia is the normal birth of a fetus in mammals. In contrast, dystocia is prolonged parturition in which (veterinary) intervention is required, otherwise fetal and/or maternal death may occur. Dystocia encompasses challenges such as malpresentation, malposition, malposture, oversized fetus, congenital fetal abnormalities, narrow birth canal, twins, uterine inertia, or fetal death [1,2]. It is highly prevalent in mammals, especially in cattle worldwide, with incidences of 1.5–6.6% in dairy cows and 4.1–8.7% in beef cows [3,4,5,6,7]. It is thought that between 17 and 40% Holstein first-parity heifers experience dystocia [3,8]. The causes of dystocia are either maternal, fetal, or both. Fetal etiologies account for 85.5% of all cases of dystocia, with the rest (14.5%) being of maternal origin [9]. Thus, the impact of dystocia extends beyond immediate obstetric concerns by affecting fertility and reducing profit [5,10]. For instance, neonates suffering from dystocia are five times more likely to die within the first 96 h of life [1]. For the cow, there is an increase in days open and the number of services per conception, both of which lower overall reproductive performance [10].

Current practices still employ traditional approaches to dystocia management, including manual manipulation and traction to achieve per vaginum delivery [11]. Success depends on timely intervention, operator skill, appropriate tools, accurate diagnosis of the cause, and the complexity of the case. Thus, a clear and concise roadmap is warranted to expedite diagnosis and resolution.

Given the need for a structured approach to dystocia that integrates advances in veterinary medicine and evidence-based practices, this paper outlines a comprehensive roadmap aimed at guiding decision-making in dystocia cases, with an overarching goal of optimizing outcomes for both cow and calf.

Drawing upon a synthesis of the literature, clinical experiences, and emerging trends, this paper delves into the key components of the proposed roadmap strategy. These components include early recognition and assessment of dystocia; utilization of diagnostic tools and technologies, such as ultrasound, fetal monitoring devices, and sensor technologies; implementation of appropriate treatment options, including medical and surgical interventions; adoption of preventive measures, such as proper nutrition and breeding management; and comprehensive postpartum care [12]. Through collaboration between veterinary professionals, farmers, and support staff, the roadmap can be effectively integrated into existing veterinary practice.

## 2. Definition and Causes of Dystocia

Dystocia, a term derived from the Greek words *dys*, meaning difficult, and *tokos*, meaning birth, refers to an abnormal or difficult labor that can compromise both maternal and fetal health [13]. In bovids, dystocia is multifactorial, influenced by genetic, nutrition, or management factors [12], although it can also occur without any explainable etiology, e.g., malpresentation (Table 1). Broadly, dystocia in cattle is categorized into maternal and fetal causes [13,14].

### 2.1. Maternal Causes

#### 2.1.1. Uterine Inertia

Primary or secondary uterine inertia is one of the main causes of dystocia due to inadequate myometrial contraction resulting in prolonged stage two of labor [15]. Multiparous animals are thought to be prone to uterine inertia compared to primiparous cows [31]. There are two types of uterine inertia: primary and secondary [13]. Primary uterine inertia in cattle often arises from excessive uterine stretching, common in multiple pregnancies, or from a defect in the myometrium that prevents effective contractions [15,16]. This condition can also result from hormonal imbalances and periparturient hypocalcemia (milk fever), a significant cause of primary uterine inertia [32]. Furthermore, hypocalcemia reduces uterine muscle tone, impairing its ability to contract properly, and is particularly prevalent in high-producing dairy cows [15,16]. Treatment involves administering calcium solutions intravenously or subcutaneously to restore normal muscle function. Prevention strategies are based on calcium-reduced feeding, administration of vitamin D, and feeding anionic salts to improve calcium mobilization postpartum. Secondary uterine inertia, on the other hand, arises when there is no progression in stage two of labor in an animal where the uterus initially contracts but becomes fatigued due to prolonged labor or obstructions [17]. Prolonged labor can be due to an oversized fetus, malpresentations, reduced pelvic size or deformities, uterine torsion, or other complications. The prolonged effort exhausts calcium reservoirs and the uterine muscles, leading to cessation of contractions [13]. Management typically involves manual assistance or surgical intervention (e.g., cesarean section) to deliver the fetus.

#### 2.1.2. Birth Canal Obstructions

Birth canal obstructions are physical impediments to uneventful expulsion of the fetus [17]. These obstructions can be categorized as pelvic fractures, strictures, and inadequate pelvic size. Fractures of the pelvis may result from trauma or congenital abnormalities and can create physical barriers within the birth canal, i.e., they narrow the inner diameter of the pelvis, making it difficult for the calf to pass through. Pelvic fractures as a cause of dystocia are usually diagnosed through physical examination. Likewise, strictures are areas of abnormal narrowing within the birth canal, which can result from scar tissue formation due to previous injuries or infections. Neoplasms and carcinoma of vaginal and urinary bladder are rare causes of pelvic abnormalities [14]. Strictures can significantly reduce the diameter of the birth canal, impeding the progress of labor. Management of strictures may involve manual dilation, surgical correction, or cesarean delivery.

The above-mentioned deviations describe the situation in the birth canal. In general, however, disproportions between the fetus and the cow (fetomaternal size disproportion) are the leading cause of dystocia in cattle, accounting for approximately 50% of all dystocia cases [15], with a greater prevalence in primiparous cows compared to multiparous cows [33]. Disparities between the size of the calf and the maternal pelvis can lead to dystocia, particularly in heifers [31] or smaller breed cows mated with larger breed bulls.

#### 2.1.3. Hormonal Imbalances

Hormone homeostasis is pivotal for the initiation, progression, and completion of eutocia, with estradiol, oxytocin, prostaglandins, and relaxin orchestrating myometrial contractions, cervical dilatation, and pelvic relaxation [19]. Dysregulation of these hormones disrupts the finely tuned parturition cascade, predisposing cows to dystocia. For instance, estradiol, synthesized by the placental–fetal unit, primes the reproductive tract for labor by upregulating uterine oxytocin receptors (OXTR) and stimulating prostaglandin synthesis [34]. During late gestation, a surge in estradiol enhances OXTR density in the myometrium, enabling effective oxytocin-driven contractions [19,34]. Low estradiol levels, often linked to placental insufficiency or nutritional deficiencies, impair OXTR expression, resulting in uncoordinated, weak contractions and delayed cervical dilation [19]. Estradiol also promotes cervical glycosaminoglycan remodeling, which is critical for cervical softening [34]. Furthermore, oxytocin, released from the posterior pituitary in pulsatile bursts, binds to OXTRs to stimulate rhythmic myometrial contractions. Exogenous oxytocin is administered to augment uterine activity in cases of primary inertia, but excessive doses or improper timing (e.g., before full cervical dilation) risk tetanic contractions, placental separation, or uterine rupture [35,36]. Continuous infusion protocols are safer than bolus dosing, as they mimic physiological response [37,38]. Prostaglandins F2α (PGF2α), secreted by the endometrium, are involved in cervical ripening and myometrial contractions via prostaglandin F receptor (FP), while prostaglandin E2 (PGE2) softens the cervix by degrading collagen fibers [39,40]. Deficient prostaglandin synthesis can delay the onset of labor and weakens contractions, often due to inadequate arachidonic acid availability or cyclooxygenase (COX) inhibition [41]. Prostaglandin analogs like cloprostenol (500 µg IM) may be used to induce labor or enhance uterine activity in cases of dystocia but require concurrent cervical readiness to avoid iatrogenic dystocia [42]. Relaxin, secreted by the corpus luteum and placenta during late gestation, helps to soften and relax the pelvic ligaments and cervix in preparation for delivery by upregulating matrix metalloproteinases (MMPs) that degrade collagen [43]. Insufficient relaxin levels can lead to a rigid pelvic ligaments and prolonged stage I labor, making it difficult for the calf to pass through [34]. While exogenous relaxin is not commercially available for cattle, nutritional strategies optimizing selenium and vitamin E (e.g., 0.3 ppm selenium in diet) enhance endogenous relaxin activity by reducing oxidative stress [44]. Proactive monitoring of prepartum hormone profiles, coupled with targeted interventions (e.g., calcium borogluconate for hypocalcemia), mitigates dystocia risk.

### 2.2. Fetal Causes

#### 2.2.1. Presentation, Position and Posture

Approximately 95% of calves are delivered in anterior presentation [24]. During the first two months of gestation, the calf has no definite polarity; by the third month, anterior and posterior presentations are equally common. From the fourth to the sixth month, most fetuses are in posterior presentation [24]. However, by the seventh month, anterior presentations become dominant [24]. Posterior presentations are more frequent in bull calves, likely due to their larger size interfering with the turning process into anterior presentation [22].

Near term, calves typically assume the anterior (cranial) longitudinal presentation, dorso-pubic position and extended posture [22]. This disposition is driven by fetal discomfort triggered by myometrial contractions, with protracted movement occurring about three days before birth [45]. Weak or dead fetuses result in inadequate signaling and an ensuing dystocia [46]. Posterior presentations are more common in twin pregnancies due to uterine crowding and a higher likelihood of uterine inertia [24]. These factors also contribute to an increased incidence of dystocia in twin and premature births.

After the righting reflex, where the fetus adopts an extended, dorso-pubic position, maintaining proper posture requires minimal fetal activity [47]. However, weak or absent fetal muscle activity, especially in stillborn calves, can lead to dystocia due to faulty posture [46]. Hypoxia can significantly affect fetal movements, either diminishing them or causing erratic limb and head movements, contributing to postural abnormalities [26]. Lateral deviation of the head is a notable postural abnormality that may arise from inadequate uterine space or may develop during late gestation rather than at birth [24].

Maldispositions refer to deviations from physiological disposition, such as breech presentations (hindquarters first), head-turned-back presentations, or transverse presentations (sideways positioning) [13,48,49]. Malpresentation can also occur in twin pregnancies [50]. These abnormal positions can obstruct the birthing process, often requiring assistance for the cow to deliver the calf. Postural irregularities of the head and limbs, including carpal flexion, lateral head deviation, and breech presentation, are relatively common occurrences during calving. Dystocia resulting from faulty fetal disposition, though less frequent, is estimated to occur in 17% to 30% of all dystocia cases, representing 2% to 4% of all births [24]. Specifically, faulty posture, position, and presentation contribute to 7.8%, 2.3%, and 8.2% of dystocia cases, respectively [4].

Abnormal fetal disposition occurs in approximately 4% of calvings, with the majority presenting uncomplicated posterior presentations (73%), followed by breech position (8%) and posterior longitudinal presentation with ventral position (1%) [46]. Among those in anterior presentation, 11% exhibit unilateral carpal or shoulder flexion, 2% display incomplete elbow extension, and 2.5% demonstrate lateral head deviation. Additionally, transverse presentation and oblique ventrovertical presentation/position occur in 1.4% and 0.6% of cases, respectively [46]. Management typically involves manual correction of the calf’s position, performed by trained professionals to minimize the risk of injury to both the calf and the cow.

#### 2.2.2. Fetomaternal Size Disproportion

Fetal oversize, or macrosomia (birth weight >45 kg in Holsteins; >40 kg in Angus), is a leading cause of dystocia, particularly in beef cattle where selective breeding for growth traits exacerbates fetopelvic disproportion [1,4,23]. Male calves are disproportionately affected due to androgen-driven fetal hypertrophy, with studies reporting a 15–20% higher birth weight compared to females, increasing the risk of shoulder impaction and uterine inertia [4,50]. Genomic analyses identify polymorphisms in insulin-like growth factor 2 (IGF2) and PLAG1 loci associated with excessive fetal growth, enabling precision breeding to mitigate risk [51]

In beef systems, breed-specific thresholds for fetal oversize vary: Charolais-sired calves average 4–6 kg heavier than Angus, correlating with a 12% higher dystocia incidence [52]. Dairy herds face similar challenges; Holstein calves sired by high-genetic-merit bulls for milk yield often exhibit birth weights exceeding maternal pelvic inlet dimensions (threshold: 13 cm vertical diameter in primiparous heifers) [23].

Preventive measures include careful sire selection to avoid large calves and managing cow nutrition to avoid excessive fetal growth and accumulation of fat in the dam’s pelvis [12].

#### 2.2.3. Congenital Abnormalities

Congenital abnormalities occurring in bovine fetuses represent a clinically significant yet underrecognized cause of dystocia due to their mechanical obstruction of the birth canal and poor prognostic outcomes [53]. Hydrocephalus, characterized by excessive accumulation of cerebrospinal fluid within the ventricular system, primarily results from three pathological mechanisms: excessive production of cerebrospinal fluid (CSF), impaired CSF absorption, or obstruction of CSF circulation, leading to abnormal accumulation within the ventricular system [54].

Likewise, *schistosomus reflexus*, a rare and fatal condition where the calf’s spine may have retroflexion of the spine between thoracic and lumbar vertebrae, malformations extending from the sacrum to the occipital bone, and exposure of thoracic/abdominal organs due to ventral body wall defects, which can also complicate the parturition or make vaginal delivery even impossible [24,55]. The genetic cause in Holstein fetus is a mutation in the *APAF1* gene (Apoptotic Protease Activating Factor 1) [55,56].

The prevalence of dystocia due to congenital abnormalities is less common compared to other causes but poses serious challenges when they do occur. Diagnosis is often made through palpation, and cesarean delivery is usually required to resolve these cases. The prognosis for calves with these conditions is generally poor, making fetotomy an option, particularly for dead calves. Prediction of dystocia is essential for optimizing calving management and minimizing its adverse consequences. Risk is primarily estimated using maternal size and fetal dimensions, particularly in cases of fetopelvic disproportion, while malpresentation remains challenging to predict due to its acute onset. In the future, hormonal imbalances may also serve as predictive markers for dystocia [33].

## 3. Implications for Cow and Calf Health

The implications of dystocia extend beyond the periparturient period. Therefore, a better understanding of these adverse outcomes is warranted for devising effective dystocia management strategies.

### 3.1. Cow Health

Dystocia triggers a cascade of physiological disruptions that predispose cows to severe metabolic and reproductive disorders, significantly impacting long-term health and productivity [57,58]. The mechanical trauma of prolonged labor or assisted extraction compromises uterine and cervical integrity, creating portals for bacterial invasion. *Escherichia coli* and *Trueperella pyogenes* exploit these breaches, colonizing the endometrium and inciting metritis within 7–10 days post-calving, and endometritis [59,60]. Dysregulated inflammation, marked by elevated serum haptoglobin, interleukin-1 (IL-1), and IL-6, delays endometrial repair and perpetuates infection, reducing conception rates. Severe dystocia can result in uterine prolapse, a life-threatening condition where the uterus is inverted and expelled outside the body, because of excessive straining during obstructive dystocia [61]. Immediate veterinary intervention is required to address this condition and prevent further complications such as hemorrhage and infection. Likewise, the incidence of retained placenta, where the placenta is not expelled within 24 h post-calving, is significantly higher in dystocia cases. This condition is primarily due to impaired prostaglandin synthesis and insufficient myometrial contractions. Incomplete detachment of placental cotyledons leaves necrotic tissue, fostering biofilm formation and endotoxin release. This elevates systemic oxidative stress, further suppressing immune function and predisposing cows to metritis and other uterine infections, further complicating postpartum recovery [62,63]. Furthermore, metritis, an infection of the uterus, is common in cows with dystocia due to the increased likelihood of uterine contamination during prolonged or assisted calving [63]. This condition can lead to systemic illness, reduced milk yield, endometritis prolonged recovery times, and decreased fertility [10].

The stress and trauma associated with dystocia perturbs fertility by delaying endometrial recovery and resumption of ovarian activity, since elevated cortisol suppresses gonadotropin-releasing hormone (GnRH) pulsatility, delaying first ovulation (>45 days postpartum) and increasing anestrus risk [64]. Therefore, cows that have experienced dystocia often take longer to return to estrus and conceive again, resulting in increased number of services per conception, prolonged calving intervals, and decreased lifetime productivity [10,65]. Additionally, cows that have experienced dystocia and associated diseases often show decreased milk production, reduced fertility, and increased culling risk [24,66]. The cost per dystocia is thought to range from $145 to $400 [67], which directly affects the profitability of dairy operations.

### 3.2. Calf Health

Dystocia not only poses risks to maternal health but also significantly impacts the well-being and survival of the newborn calf as well as long-term health and development. Therefore, understanding the implications of dystocia on calf health is essential for implementing appropriate management strategies and interventions to mitigate adverse outcomes.

A primary concern is higher mortality after dystocia [24,32,68]. Studies have indicated that calves born after dystocia have up to a fivefold increase in mortality rates compared to those from normal births [1]. In a previous study monitoring 285 calvings, dystocia was associated with a 14.3% mortality rate in calves [69]. similar findings were reported in ewes [70]. This heightened mortality risk can be attributed to a variety of factors, including physical trauma during birth and the increased likelihood of delayed veterinary intervention in difficult calvings.

Dystocia can also result in behavioral and neurological complications in calves [71]. Hypoxia, a common consequence of dystocia, leads to insufficient oxygen supply and can depress central nervous system function, resulting in delayed behavioral responses and impaired motor functions, including compromised respiratory efficiency [50]. These neurological impairments may hinder the calf’s ability to stand and nurse shortly after birth, which are critical behaviors for early survival and optimal growth [71].

Dystocia can disrupt the normal transfer of passive immunity to the newborn calves, e.g., due to reduced colostrum intake, leaving them more susceptible to infections, diseases, and an increased risk mortality [32,66,68]. Additionally, prolonged labor or thoracic compression during birth can lead to respiratory distress, risking hypoxia and respiratory failure if not managed swiftly [50]. Dystocia may also disrupt colostrum antibody transfer, weakening immune function in the neonatal period and increasing the likelihood of bacterial and viral infections [68]. This immune compromise contributes to higher morbidity and mortality rates, which can impact herd health [66]. Likewise, dystocia survivors may exhibit impaired growth and development compared to calves born from uncomplicated deliveries [72]. The physiological stress of dystocia, coupled with potential health complications, can hinder optimal growth during the neonatal period, leading to reduced weight gain and delayed weaning [11]. Thus, prompt intervention and careful management of dystocia are essential to mitigate these long-term impacts.

## 4. Current Practices in Dystocia Management

### 4.1. Non-Surgical Approaches

Traditional approaches to managing dystocia primarily involve close monitoring of the labor process and timely intervention when complications arise. Interventions may include manual assistance, the use of calving aids, or, in more severe cases, surgical procedures [5,11] (Table 2). Manual assistance with or without instruments involves repositioning the calf, applying traction, or assisting in the delivery using one’s hand [11,21]. This approach requires skill and experience to minimize trauma to both the cow and the calf. Commonly used obstetric tools include obstetric chains and handles which are used to apply controlled traction to the calf’s limbs to assist in delivery [11]. Obstetrical instruments have been used since decades, e.g., Caemmerer torsion fork and Kuhn’s crutch. More recently developed tools combine these instruments (e.g., “GYN-stick”). Calf puller (Calf Jacks), also known as fetal extractors, provide mechanical assistance to exert greater force during delivery [21]. While useful, improper use can lead to excessive force and injury. Proper sanitation and lubrication are critical to prevent infections and ensure smoother manipulations [73]. The primary objective of all intervention is to ensure the safe delivery of the calf while minimizing the risk of harm to the cow. These methods heavily rely on the experience and clinical judgment of the attending veterinarian or farm staff, and when executed appropriately, they can be highly effective. However, the success of these interventions is often contingent upon early recognition of labor complications and the skill of the personnel involved.

Hormonal treatments for the management of uterine inertia and dystocia have been described above (Section 2.1.3). In brief, oxytocin stimulates the smooth muscles of the uterus, causing contractions that help expel the fetus during labor, especially when uterine contractions are weak or insufficient [35,78]. The dosage of oxytocin must be carefully calculated based on the weight of the cow and the specific circumstances of dystocia [35]. The response to oxytocin is usually rapid, with uterine contractions often resuming within minutes of administration. Continuous monitoring of the cow after administering oxytocin is essential to ensure that the contractions are strong enough to aid in delivery but not so intense as to cause harm, mainly uterine rupture, fetal distress, and maternal exhaustion [36].

Administering calcium along with oxytocin can help enhance uterine contractions [18]. Other supplements, such as glucose or magnesium, may also be used depending on the specific metabolic status of the cow as they have critical role in the contraction of myometrium [18]. Furthermore, providing supportive care, such as maintaining hydration and energy levels, is important when using hormonal treatments. Ensuring the cow is in a comfortable, stress-free environment can also aid in the effectiveness of oxytocin.

### 4.2. Surgical Approaches

#### 4.2.1. Cesarean Section

In cases where manual and tool-assisted interventions fail, a cesarean section (C-section) may be necessary [18,74]. This surgical approach is typically reserved for severe dystocia cases and requires a skilled veterinarian. If the cow or calf shows signs of distress, such as prolonged labor with no progress, decreased fetal heart rate, or maternal exhaustion, C-section may be necessary to expedite delivery and prevent further complications [5]. Also, certain anatomic abnormalities in either the cow or the calf, such as uterine torsion or severe skeletal malformations, may necessitate a cesarean section due to the inability to deliver the calf vaginally [3,18,24,74]. Veterinary surgeons perform C-sections under general anesthesia, with careful attention to maternal and fetal well-being [22]. The procedure is typically performed via a left flank approach (95% of cases) to minimize rumen displacement, with the cow restrained in standing position under regional anesthesia (e.g., inverted L block: 100–150 mL 2% lidocaine). For recumbent cows, general anesthesia using xylazine (0.05 mg/kg IV) followed by ketamine (2 mg/kg IV) provides safe induction. A 20–25 cm vertical incision is made parallel to the last rib, followed by exteriorization of the uterus and a 10–15 cm hysterotomy to extract the calf [79].

For cow’s pain management, preoperative analgesia includes meloxicam (0.5 mg/kg IV) to inhibit prostaglandin synthesis, reducing intraoperative stress and improving post-operation fertility [80]. For the calf, resuscitation protocols include oxygen insufflation (5 L/min via nasal cannula) and nalbuphine (0.1 mg/kg IM) for hypoxia-induced discomfort [81].

#### 4.2.2. Episiotomy

Episiotomy, a surgical incision of the perineal body and vestibular mucosa, is indicated in cattle to resolve dystocia caused by severe vaginal stenosis, obstructive scar tissue, or relative fetal oversize that prevents safe vaginal delivery [82]. The procedure is typically reserved for cases where manual dilation fails or risks extensive soft tissue trauma, such as primiparous heifers with rigid perineal structures or cows with prior traumatic vulvar injuries [82]. Episiotomies are performed under local anesthesia and require careful postoperative management to prevent infection and promote healing [18]. In addition, it can be applied in cases where the calf is dead [82]. According to Behera et al. [82], the animal is restrained and stabilized with dextrose normal saline, and the perineum is disinfected with 1% KMnO_4_. Aseptic preparation follows, with epidural anesthesia administered (5 mL 2% lignocaine at the first intercoccygeal space). A 4-inch incision is made at the right dorso-lateral vulva (1 o’clock position) after lubricating the birth canal with 2% sodium carboxymethylcellulose. The fetus, positioned in anterior longitudinal, dorsosacral orientation with flexed forelimbs, is delivered using an obstetrical snare and simple traction. Perineal muscles are sutured with No. 2 catgut, and the skin is closed with No. 3 silk in a cross-mattress pattern. The placenta is expelled within 2 h. Postoperative treatment includes meloxicam (0.5 mg/kg IM), ceftriaxone/tazobactam (3375 mg IM), vitamin B-complex (10 mL IM), and intrauterine therapy with levofloxacin, ornidazole, and vitamin E for five days. Skin sutures are removed on day 14, and the animal recovers uneventfully.

#### 4.2.3. Fetotomy

Fetotomy, the surgical dissection and removal of a deceased or malformed fetus, is a critical intervention for resolving dystocia when vaginal delivery is obstructed and cesarean section poses greater risks to the dam [18,22]. Indications include emphysematous fetuses, irreparable malformations (e.g., schistosomus reflexus, severe arthrogryposis), or cases where prolonged dystocia has compromised fetal viability [83]. The procedure requires advanced technical skill to minimize maternal trauma, with success rates exceeding 80% when performed by experienced practitioners. However, the duration of the procedure can be variable and challenging to predict, as the number of cuts required is not always readily apparent. The decision to perform a C-section or fetotomy depends, among other factors, on the expected duration of the procedure, influenced by the position of the calf, malformations, width of the birth canal, and whether a total or partial fetotomy is required to remove the fetus [11,21].

Dystocia causes significant discomfort and distress in cattle, requiring appropriate pain management strategies to enhance animal welfare, reduce stress responses, and support recovery [84]. Pain management strategies, such as nonsteroidal anti-inflammatory drugs (NSAIDs), can help alleviate pain and reduce stress during labor and recovery [85]. NSAIDs are the cornerstone of pharmacological pain management in dystocia cases due to their anti-inflammatory, analgesic, and antipyretic properties. Meloxicam (0.5 mg/kg SC or IV, single dose) is a commonly used NSAIDs in bovine practice, since it offers long-lasting analgesia with fewer gastrointestinal side effects compared to other NSAIDs [80]. Flunixin meglumine (2.2 mg/kg IV, repeated every 24 h if necessary) is a potent cyclooxygenase (COX)-1 inhibitor that provides effective visceral pain relief but may have adverse renal and gastrointestinal effects and may increase the risk of stillbirth [86]. Furthermore, ketoprofen (3 mg/kg IM or IV, up to 3 days), a short-acting NSAID with minimal withdrawal periods but less potent anti-inflammatory action than meloxicam or flunixin, may confer similar analgesic effect compared to meloxicam [87]. Despite the powerful analgesic effects, NSAIDs should be used with caution in cattle with renal dysfunction or dehydration, as they can reduce renal perfusion and exacerbate kidney injury. Prolonged use may also lead to gastrointestinal ulceration or hemorrhage, increasing the risk of complications. Additionally, co-administration with corticosteroids should be avoided due to the heightened likelihood of gastrointestinal damage and adverse systemic effects, as reported in several species, including humans.

Local anesthetics play a pivotal role in regional pain control. Epidural anesthesia using lidocaine (0.5 mg/kg) injected into the sacrococcygeal space reduces straining and perineal pain during obstetrical manipulations, while pudendal nerve blocks with 2% lidocaine (10–15 mL per side) desensitize the vulvar and vaginal regions for procedures such as episiotomy [88]. Care must be taken to avoid exceeding the maximum lidocaine dose of 10 mg/kg to prevent neurotoxic effects such as lethargy or seizures. Opioids provide short-term analgesia (2–4 h) but are infrequently used in cattle due to regulatory restrictions and limited availability.

Following dystocia management, careful post-treatment care is essential to ensure both maternal recovery and neonatal survival. Effective pain management is a priority, with NSAIDs recommended for 24–48 h post-intervention to reduce inflammation and alleviate discomfort. This helps improve the cow’s overall well-being and facilitates a smoother recovery. Close monitoring for potential complications is crucial, including signs of uterine prolapse, hemorrhage, or retained fetal membranes, which may require prompt veterinary intervention. Supportive therapy plays a vital role in recovery, particularly in cases of prolonged dystocia or dehydration. Administration of intravenous fluids, such as Ringer’s lactate or dextrose-saline solution, helps restore hydration and electrolyte balance, promoting systemic stability. Additionally, antimicrobial prophylaxis is recommended in high-risk cases to prevent postpartum infections. Broad-spectrum antibiotics, such as ceftiofur or oxytetracycline, can be administered to mitigate the risk of bacterial infections, particularly in cows with traumatic birth injuries or retained fetal membranes [1]. Neonatal management is equally critical, as calves born from dystocia cases are at an increased risk of hypoxia, fractures, or neurological deficits. Immediate postnatal assessment should focus on detecting signs of respiratory distress, musculoskeletal injuries, or failure to suckle. In cases where the calf is weak or unable to nurse effectively, assistance with colostrum intake is necessary to ensure passive transfer of immunity and enhance survival outcomes.

### 4.3. Limitations, Challenges, and Drawbacks

Managing dystocia in cattle presents several challenges that require prompt and effective solutions. Early detection of dystocia is critical as delays can exacerbate both maternal and fetal distress. Training farm staff and veterinarians to identify signs of dystocia and to initiate timely and effective interventions is essential for minimizing adverse outcomes. Advanced diagnostic tools like ultrasonography and fetal monitoring devices [89,90] are vital for early detection and management of dystocia [91]. However, these tools may not be readily available in all settings. Manual and tool-assisted interventions heavily rely on the skill and experience of the handler. Inadequate training or lack of experience can lead to improper use of tools or practices, increasing the risk of injury to both the cow and the calf.

Resource constraints represent another significant challenge in the management of dystocia. These limitations can span from financial constraints to the availability of equipment, the training of staff, to the call for veterinary services. The costs associated with regular veterinary care, advanced diagnostic tools, and necessary interventions can be prohibitive for some farms, particularly for small-scale or subsistence farmers. Solutions to address these challenges include group purchasing programs for equipment, shared access to veterinary services among small farms, or government subsidies for essential livestock services. Furthermore, education and training programs can equip farmers and farm workers with the knowledge and skills to effectively manage dystocia even in resource-limited settings.

## 5. Need for a Structured Roadmap Strategy

A structured roadmap strategy could provide a clear protocol for monitoring labor and identifying signs of dystocia, as well as guidelines for when and how to intervene. This strategy could incorporate the use of advanced diagnostic tools and monitoring devices, where available, to improve early detection of dystocia. It could also include training programs for farmers and farm staff to improve their skills in managing difficult births [13]. In addition, a structured roadmap strategy could also address resource constraints by outlining cost-effective approaches to dystocia management and strategies for improving access to necessary resources and services.

## 6. Components of the Dystocia Management Roadmap

### 6.1. Early Recognition and Assessment

#### 6.1.1. Importance of Early Detection

The first component of the dystocia management roadmap involves accurate detection of the onset of parturition and the early recognition and assessment of dystocia. This is a critical step as early identification can significantly improve the chances of successful intervention and minimize harm to both the cow and the calf. Predicting labor by observation, however, is a challenge [92]. The longer a cow is in distress from a difficult birth, the greater the risk to both her and the calf’s health and survival [3,50]. On the other hand, early but unnecessary interventions due to inaccurate assessment of parturition/dystocia may also lead to an avoidable disturbed parturition.

Early signs of dystocia in cows include prolonged labor, abnormal behavior such as excessive restlessness or distress, and distinct changes in movement patterns before calving [93]. Research has shown that cows with dystocia exhibit specific behavioral alterations as early as 24 h before parturition. For instance, cows with dystocia transition from standing to lying positions more frequently than those with eutocia (10.9 ± 0.7 vs. 8.3 ± 0.7 bouts/day) [12]. Additionally, in the hours leading up to calving, affected cows show an increase in tail contractions and raised tail position, along with a reduction in eating behavior and rumination time [94].

Furthermore, daily standing times tend to peak around calving (14.4 h) compared to the precalving period (12.3 h), with a significant increase in standing bouts on the day of parturition (21.8 bouts vs. pre- and postcalving averages of 11.7 and 13.1, respectively) [95]. Changes in feed and water intake have also been observed in cows experiencing dystocia, with a notable decrease in water consumption 24 h before calving (22.4 ± 4.4 vs. 36.2 ± 4.4 kg/day) and a compensatory increase post-calving [12]. Additionally, dry matter intake and standing bouts before calving are among the most reliable predictors of dystocia [12].

Parity also plays a role in pre-calving behavior, as primiparous cows exhibit increased physical activity, including more postural transitions (37.7 ± 1.2 vs. 27.6 ± 0.7 transitions/day in multiparous cows) and greater walking distance in the days before calving [96]. These behavioral changes, particularly increased restlessness, frequent transitions between standing and lying, and reduced feeding and rumination time, provide valuable indicators for early dystocia detection and intervention.

Advances in technology, such as electronic calving monitors and wearable sensors, can aid in the early detection of parturition or dystocia [76,77]. These devices can monitor various parameters, including uterine contractions, calf movement, and cow behavior, providing alerts when abnormalities are detected. Some systems can be applied into the cow’s vagina or at the tail as a warning system, e.g., if the device is expelled or the tail moves with a defined frequency [97,98].

New approaches use sensor technologies for an early detection of the onset of parturition. In these systems, algorithm process data from accelerometers and predict the onset of calving [99]. The accuracy of these systems, however, is still limited and needs to be improved. Further research is required to show if deviations in animal behavior that predict dystocia can be monitored by sensor technologies.

#### 6.1.2. Signs of Dystocia

While there are no clear boundaries between dystocia and eutocia, dystocia is defined as a prolonged first or second stage of labor requiring assistance for delivery [13]. Providing guidelines based on the progress and duration of labor can help farmers and veterinarians determine when to intervene, what actions to take, and what resources are needed [50]. Normally, the first stage of labor, which involves cervical dilation, lasts between 2 and 6 h [100]. If this stage exceeds 8 h without progression to the second stage, which is the active pushing and delivery, it may indicate dystocia [100]. The second stage of labor in cows typically spans from 0.5 to 4 h; according to intervention guidelines, assistance is recommended if the second stage exceeds 2 h, and earlier if progress is not normal [100]. Nevertheless, it is noteworthy that a calf can endure the second stage of labor for up to 8 h [49,100]. Prolonged pushing without visible progress, or the appearance of abnormal presentations of the calf, can signal dystocia. The presence of abnormal vaginal discharge, such as a foul-smelling or bloody fluid, can be an indication of uterine infection or other complications associated with dystocia [100,101]. In addition, signs of maternal distress, including excessive restlessness, signs of pain, and exhaustion, may suggest dystocia and the need for intervention [11].

#### 6.1.3. Training and Preparedness

Recognizing these signs requires a keen eye and understanding of the normal birthing process and detecting signs for dystocia accurately. This underscores the importance of training for farm staff. For instance, regular training sessions and workshops should be conducted to educate farm personnel about the signs of dystocia, appropriate responses, and the importance of timely intervention. Training should include both theoretical knowledge and practical skills, such as palpation techniques and the use of obstetric tools, such as chains and handles [13,102]. Also, establishing Standard Operating Procedures (SOPs) for the detection and management of dystocia and when to call a veterinarian can help streamline the process and ensure consistent responses [103]. Farms should be equipped with necessary tools and supplies for managing dystocia, such as obstetric chains, calf jacks, lubricants, and appropriate medications [11]. Having a well-prepared calving area with clean, easily accessible facilities can also facilitate timely interventions.

Using recent technologies such as telemedicine can offer a potential solution to bridge the gap in veterinary care access. Through telemedicine, farm personnel can consult with veterinary specialists remotely, sharing real-time data and images to receive guidance on managing dystocia cases. This approach can be particularly valuable in areas where in-person veterinary services are limited, allowing for timely expert advice and decision-making.

#### 6.1.4. Scoring Systems

Implementing scoring systems, such as the calving ease score from 1 to 5, can help standardize the assessment of dystocia risk and management [104,105]. These scoring systems include various factors, such as calf presentation, cow body condition, and previous calving history, to predict the likelihood of dystocia and guide decision-making. These systems vary in complexity, from simple binary classifications to more detailed multi-point scales and advanced predictive models (Table 3). The choice of scoring method significantly impacts the reported incidence of dystocia, data interpretation, and subsequent management strategies [105,106,107]. The most widely recognized system for dystocia classification is the 5-point calving ease score, initially proposed by Berger al. (1994). This system categorizes calving difficulty as follows: score 1 = no problem, score 2 = slight problem, score 3 = need for assistance, score 4 = considerable force is required, to score 5 extreme difficulty during the calving process [105]. This system is frequently used in research and herd management due to its ability to distinguish varying degrees of difficulty and guide intervention strategies. An alternative method is the binary classification system, which simplifies dystocia assessment into two categories: eutocia (normal calving) and dystocia (any difficulty requiring assistance) [106]. While practical for field use, this system lacks the granularity needed for detailed epidemiological studies and management decisions. Advanced machine learning algorithms, such as random forest (RF) and boosted trees (BT) have been developed to predict dystocia in dairy heifers and cows. These models analyze multiple variables, including cow parity, body condition, fetal size, and labor duration, to improve prediction accuracy [108].While boosted trees tend to exhibit better sensitivity in detecting difficult calvings, random forest models generally achieve higher overall accuracy. However, neither model is currently considered practical for field application due to limitations in accurately detecting difficult calvings and propensity for generating false alarms [106,109]. Beyond scoring systems, alternative dystocia prediction methods have been investigated. Fetal bone thickness is assessed using transrectal ultrasonography in late-term heifers and cows, and lower metacarpal/metatarsal index (MCTI) would indicate association with dystocia risk [110].

A crucial consideration when using dystocia scoring systems is the degree of difficulty included in the definition of dystocia. Variations in classification criteria can lead to significant discrepancies in the reported dystocia incidence. For example, Roche et al. [106] reported an incidence of 4.3%, whereas Holm et al. [108] reported 33.5%, largely due to differences in the definition used—only score 3 was considered dystocia in the former, while scores 2 and 3 were included in the latter. Such discrepancies emphasize the need for standardized classification criteria in both research and farm-level management.

Despite the availability of structured scoring systems, clinical decision-making remains inherently subjective. The decision to use a calf puller, perform surgery, or intervene manually often depends on the experience of the farmer or veterinarian, farm resources, and time constraints. Standardized guidelines and training programs may help reduce variability in dystocia management decisions [33].

#### 6.1.5. Biochemical and Hematological Analyses and Endocrine Testing

Biochemical and hematological analyses offer complementary diagnostic information in cases of dystocia and for postpartum and postnatum period, respectively. Blood tests assessing electrolyte levels, blood gases, and metabolic parameters provide insights into maternal health status and identify conditions such as hypocalcemia or metabolic acidosis, which may contribute to dystocia [116,117]. Additionally, assessment of maternal and fetal blood samples facilitates the detection of hematological abnormalities or infectious diseases, guiding appropriate treatment strategies [118].

Endocrine testing, including hormonal assays and pregnancy-specific markers, provides valuable diagnostic information in cases of dystocia. Measurement of hormone levels, such as progesterone or cortisol, enables assessment of maternal endocrine status and may identify hormonal imbalances contributing to dystocia [119,120]. Additionally, pregnancy-specific markers, such as pregnancy-associated glycoproteins (PAGs), confirm fetal viability and gestational age, guiding management decisions in dystocia cases [25].

### 6.2. Treatment Options and Preventive Measures

Another important component of the dystocia management roadmap involves determining the most appropriate treatment options. Depending on the severity and cause of dystocia, this may involve medical interventions, surgical interventions, or a combination of both, as described above. Preventing dystocia is a critical component of herd health management and involves implementing a variety of strategies aimed at reducing the incidence of difficult births [121]. Overall, dystocia levels should be kept within acceptable level which is <15% for heifers and <5% for cows [122]. Preventive measures focus on genetic selection, nutritional management, proper animal husbandry, and regular health monitoring to minimize risk factors associated with dystocia [3,12,104].

#### 6.2.1. Genetic Selection and Breeding Management

One of the most effective long-term strategies for preventing dystocia is selecting bulls and cows with traits favorable for calving ease [29,30]. Genetic selection tools, such as Estimated Breeding Values (EBVs) or Expected Progeny Differences (EPDs), help identify animals with a lower propensity for dystocia [30,52]. Breeding programs should prioritize traits such as pelvic size, calf birth weight, and overall body conformation to enhance calving success [123]. For instance, the use of sexed semen resulted in a 28% reduction in difficult births among heifers and a 64% reduction among cows [104]. Implementing crossbreeding programs can also reduce dystocia incidence by introducing breeds with characteristics conducive to easier calving, such as smaller calf size at birth [124]. reeding management includes also the selection of heifers rather according to weight and body condition than to age.

#### 6.2.2. Nutritional Management

Feeding balanced diets tailored to the physiological demands of gestation is critical for optimizing fetal development, maternal health and calving outcomes. A nutritionally complete ration must provide adequate energy, protein, vitamins (A, D, E), and minerals, with particular emphasis on calcium (Ca) and phosphorus (P) to sustain myometrial contractility and prevent hypocalcemia-induced uterine inertia [2,125,126]. Excessive energy intake, particularly during the final trimester, predisposes cows to fetal macrosomia by upregulating placental insulin-like growth factor 1 (IGF-1), increasing dystocia risk in primiparous heifers [3,12]. Conversely, protein or energy deficits impair pelvic ligament elasticity and skeletal muscle tone, reducing expulsion force during stage II labor [126]. Calcium homeostasis is especially vital in late gestation; suboptimal dietary Ca or an inverted Ca:P ratio disrupts neuromuscular function, elevating the risk of primary uterine inertia [126,127]. Strategic supplementation of anionic salts (e.g., MgSO_4_, NH_4_Cl) in the prepartum diet induces mild metabolic acidosis, enhancing Ca mobilization from bone reserves and reducing clinical hypocalcemia incidence by 40% [126]. Trace minerals such as selenium (0.3 ppm) and zinc (40 ppm) further support antioxidant defenses and cervical collagen remodeling, respectively, ensuring efficient parturition [125]. Close monitoring of body condition score (BCS) is essential: cows with BCS > 3.5 (5-point scale) at calving face a 1.7-fold higher dystocia risk due to pelvic fat infiltration, while BCS < 2.5 correlates with weak contractions from depleted glycogen reserves [5,128]. Regular ration analysis and adjustments during the last 60 days of gestation, coupled with access to clean water and high-fiber forages, are key to maintaining metabolic equilibrium and minimizing calving complications.

#### 6.2.3. Proper Animal Husbandry Practices and Data Recording

Routine health monitoring and veterinary check-ups help detect and manage conditions that could predispose cows to dystocia. Regularly assessing BCS, monitoring for signs of metabolic disorders, and ensuring timely intervention for health issues are vital components of preventive care [3,12,129]. Likewise, providing adequate exercise and maintaining a stress-free environment for pregnant cows can improve their physical condition and readiness for calving. Adequate space, proper bedding, and minimizing environmental stressors, e.g., avoiding re-grouping close to parturition, contribute to overall well-being, and reduce the risk of dystocia [130].

Keeping detailed records of breeding, calving outcomes, and any incidences of dystocia helps identify patterns and risk factors within the herd. This data-driven approach enables informed decision-making and the development of targeted prevention strategies.

By systematically following this roadmap, veterinary professionals and farm personnel can ensure that each case of dystocia is managed efficiently and effectively, minimizing risks and promoting the health and well-being of both cows and calves.

## 7. Logical Application of the Dystocia Management Roadmap

**Figure 1:**
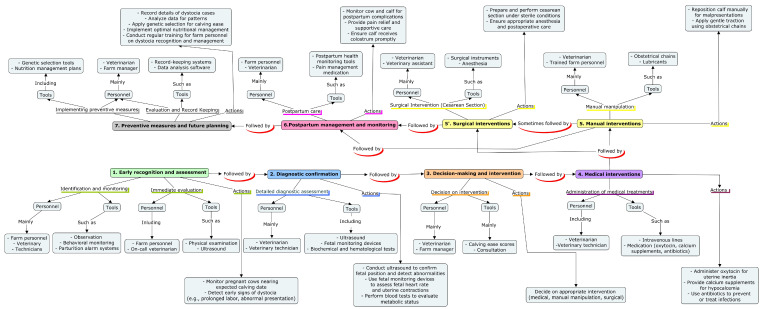
Strategic roadmap for comprehensive dystocia management. This map depicts the logical application of the strategy in every single case of dystocia. It details the steps taken, the individuals involved, and the tools used. The progression from the initial identification of dystocia, through the use of diagnostic tools and technologies, the selection and application of treatment options (both medical and surgical interventions), and finally the implementation of preventive measures to reduce future incidence of dystocia are clearly shown.

**Figure 2:**
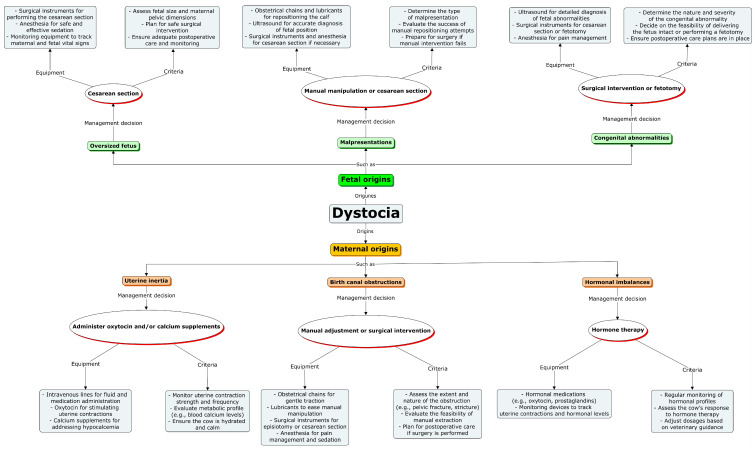
Conceptual roadmap for dystocia management. It illustrates a comprehensive roadmap for the management of various forms of dystocia, including fetal and maternal dystocia. The roadmap is divided into three key stages: management decision, used equipment, and criteria of decision. Additionally, the use of various diagnostic and treatment tools is highlighted.

A comprehensive roadmap for managing various forms of dystocia, encompassing both fetal and maternal causes, is outlined in Figure 1 and Figure 2 and Table 4.

## 8. Conclusions

Dystocia in cows significantly impacts both maternal and fetal health, with notable economic consequences. Effective management is crucial, involving timely recognition, accurate diagnosis, and appropriate interventions for various maternal and fetal causes. A structured roadmap strategy enhances decision-making and ensures the availability of necessary tools and skilled personnel. The dystocia management roadmap provides a systematic approach to improving health outcomes and reducing economic losses. It emphasizes continuous training, advanced diagnostics, and preventive measures. Veterinary professionals and farm managers should prioritize training, resource investment, collaboration, research, and preventive strategies. Adopting this roadmap can improve cow and calf health, enhance productivity, and promote sustainable livestock management practices.

## Figures and Tables

**Table 1:** Comprehensive overview of causes and contributing factors of dystocia in cattle.

Cause	Contributing Factors	Description	References
Maternal factors	Uterine inertia	Failure of the uterus to contract effectively, often due to exhaustion, nutritional imbalances, or hormone deficiencies.	[13,15,16]
	Narrow pelvic canal	Physical obstruction caused by a narrow pelvis, common in heifers or cows with pelvic fracture or certain conformation issues.	[14,17]
	Uterine torsion	Twisting of the uterus, which can obstruct the passage of the fetus and requires corrective manipulation or surgical action.	[18]
	Cervical incompetence	Inability of the cervix to dilate fully, leading to prolonged labor and increased risk of fetal distress.	[1,18]
	Hormonal imbalance	Deficiencies or surpluses in hormones such as oxytocin or calcium, leading to weak contractions or poor cervical dilation.	[19,20]
	Prolonged gestation	Extended pregnancy that can lead to oversized fetuses, creating difficulty in passage through the birth canal.	[1]
Fetal factors	Malpresentation	Abnormal orientation of the fetus, such as a breech position where the hindquarters present first, complicating delivery.	[21,22]
	Malposition	Deviation from the normal alignment, such as lateral head positioning, making delivery more difficult.	[21,22]
	Malposture	Abnormal positioning of limbs, such as flexed legs, preventing smooth passage through the birth canal.	[21,22]
	Fetal oversize	Fetus is too large relative to the dam’s pelvis, often due to breed differences or prolonged gestation.	[23]
	Congenital abnormalities	Fetal deformities (e.g., hydrocephalus, ankylosis) that can prevent normal passage through the birth canal.	[24]
	Multiple fetuses	Twin or triplet pregnancies can cause positioning issues, uterine inertia, or space limitations within the uterus.	[1,2,25]
	Fetal hypoxia	Oxygen deprivation during labor can result in erratic fetal movements, complicating positioning and delivery.	[26]
Environmental factors	Heat stress	High ambient temperatures can increase risk of uterine inertia and labor complications, particularly in late pregnancy.	[27]
	Nutritional deficiency	Inadequate intake of nutrients, especially calcium, can predispose cows to uterine inertia and weak contractions.	[12]
	Housing and Flooring	Slippery or rough flooring in the calving area may hinder the cow’s ability to push effectively during labor.	[28]
Management factors	Inexperienced assistance	Untrained personnel may mismanage labor or improperly assist in calving, increasing risk of complications.	-
	Timing of Intervention	Delay or premature intervention in labor can either miss the optimal window for natural delivery or add unnecessary stress.	-
	Over-conditioning	Excessive body fat, especially in older cows, can lead to increased risk of narrow pelvis and metabolic issues during labor.	[3,12]
	Breeding strategy	Selective breeding for larger offspring can result in fetal oversize and calving complications, especially in heifers.	[29,30]

**Table 2:** Interventions for dystocia management in cattle.

Intervention Type	Description	Advantages	Limitations	References
Manual assistance	Repositioning the calf, applying traction, or manually guiding the calf through the birth canal; typically performed by trained personnel.	Minimizes use of equipment; allows immediate response; low cost.	Requires skilled personnel; risk of injury to calf and cow; risk of infection without sanitation.	[73]
Calving aids	Use of devices such as ropes, chains, and handles to provide controlled traction and assist calf delivery.	Provides controlled force; can facilitate faster delivery; effective in minor cases.	Risk of trauma with excessive force; may require additional training to use properly.	[1]
Fetotomy	Surgical removal of parts of the calf to facilitate extraction when the calf cannot be delivered intact.	Avoids cesarean section in certain severe cases; can prevent further maternal distress.	Invasive; risk of internal injury; requires high skill level and specialized tools.	[11,21]
cesarean section	Surgical procedure to deliver the calf through an incision in the cow’s abdomen and uterus, typically used when other methods fail.	Essential in severe dystocia; preserves the life of the cow and sometimes the calf; high success rate.	High cost; requires veterinary expertise; increased risk of infection and recovery time.	[18,74]
Hormonal therapy	Administration of hormones such as prostaglandins or corticosteroids to enhance labor, particularly in cases of hormonal imbalance in maternal origin.	Can restore hormonal balance; may help induce labor.	Not suitable for all dystocia cases; requires veterinary oversight; timing is critical.	[19]
Ultrasound-guided opu	Diagnostic and interventional ultrasound used to assess fetal position and identify abnormalities in real-time; can guide other interventions.	Provides visual aid; non-invasive; enhances decision-making accuracy.	Requires specialized equipment and training; not universally accessible; high equipment cost.	[1]
Analgesia and sedation	Administration of analgesics or sedatives to alleviate pain and stress during interventions, allowing safer manipulation.	Reduces pain and stress; improves animal welfare; can ease difficult manipulations.	May prolong labor if dosage is too high; requires veterinary oversight; potential drug residues in milk.	[18]
Episiotomy	Surgical incision in the perineal area to enlarge the birth canal when soft tissue obstruction is evident or anticipated.	Prevents soft tissue tearing; facilitates delivery of large or difficult calves.	Requires post-operative care; risk of infection; invasive procedure.	[18]
Hydrotherapy	Use of warm water baths to relax the pelvic muscles and potentially ease delivery in minor dystocia cases.	Non-invasive; enhances muscle relaxation; improves comfort for the cow.	Limited effectiveness in severe cases; requires water access and sanitation measures.	[75]
Fetal monitoring devices	Use of devices to monitor fetal heart rate and detect distress, enabling timely intervention if complications arise.	Real-time monitoring; allows early detection of distress; can inform need for immediate intervention.	Requires equipment and training; may not be available in all settings; high cost.	[76,77]
Education and training	Continuous training for farm staff and veterinarians to recognize signs of dystocia early and perform interventions effectively.	Improves overall dystocia management; enhances animal welfare; cost-effective long-term.	Requires time and resources; variable effectiveness based on personnel’s skills and experience.	-
Resource-sharing programs	Collaborative programs among small farms for sharing veterinary services, equipment, and expertise, addressing financial and resource constraints.	Cost-effective for small-scale farms; improves access to essential services.	Requires coordination among farms; limited availability in some regions; may not address urgent needs.	-
Government subsidies	Financial support from government to make critical dystocia management resources more affordable, especially for small-scale farmers.	Increases accessibility to veterinary care and equipment; supports animal health on a larger scale.	Requires consistent funding; may not cover all resources; limited to specific regions.	-

**Table 3:** Overview of dystocia scoring systems reported in the scientific literature [33].

Scale	Scoring Definition	Dystocia Definition	Dystocia Incidence	Study
2-point	1: Unassisted births 2: Assisted, including all births assisted by manual pull, chain pull, jack, or cesarean section	Degree 2	28.8%	[111]
3-point	1: No assistance 2: Assistance required 3: Surgical intervention required	Degree 2–3	33.5%	[108]
3-point	1: No assistance 2: Easy pull 3: Moderate to hard pull	Degree 3	4.3%	[106]
4-point	1: No assistance 2: Farmer assistance without/with malpresentation of the calf 3: Veterinarian assistance without/with malpresentation of the calf 4: Cesarean section	Degree 2–4	33% (H) 12.8% (C)	[112]
4-point	1: No assistance 2: Little assistance with one person 3: Heavy assistance with one person or a mechanical puller 4: Difficult birth with veterinary assistance	Degree 3–4	41% (H) 10% (C)	[113]
5-point	1: No assistance 2: Slight problem (assistance for <15 min) 3: Needed assistance (assistance for >15 min with moderate difficulty of extraction) 4: Considerable force used 5: Extreme difficulty or needed veterinary assistance	Degree 3–5	27%	[114]
5-point	1: No problem 2: Slight problem 3: Needed assistance 4: Considerable force 5: Extreme difficulty	Degree 3–5	8.2%	[107]
5-point	1: No assistance needed 2: Easy pull (one person with minimal effort) 3: Moderate pull (one person with moderate effort) 4: Hard pull (one person with considerable effort or two people) 5: Mechanical extraction or cesarean section	Degree 4–5	35% (H) 6% (C)	[115]

**Table 4:** Summary of factors affecting fetal position and outcome in dystocia.

Factor	Description	Impact on Fetal Positioning	Associated Risks	Management Strategies
Maternal pelvic shape	Variations in pelvic dimensions and shape can restrict fetal movement.	Increased likelihood of fetal malposition and malposture.	High risk of prolonged labor and difficult delivery; risk of calf and cow injury.	Regular pelvic measurements in breeding animals; selection of sires with smaller birth weights for at-risk cows.
Breed and genetics	Certain breeds (e.g., large-framed breeds) have genetic predispositions to dystocia.	Increased incidence of malpositioned fetuses in large-breed cattle.	Risk of fetopelvic disproportion; higher likelihood of needing assisted delivery or cesarean section.	Selective breeding to reduce dystocia prevalence; monitoring high-risk breeds; advanced planning for large breeds.
Maternal age and parity	Age and number of previous pregnancies affect uterine and pelvic flexibility.	Higher incidence of malpositions in young and first-time heifers.	Increased risk of soft tissue trauma in young cows; higher incidence of fetal stress and mortality.	Close monitoring during labor; additional assistance and support for younger cows or those in first parity.
Nutritional status	Nutrient imbalances can affect uterine tone and fetal growth.	Poor fetal alignment due to uterine muscle tone issues.	Risk of fetal oversize or underdevelopment; weak contractions leading to prolonged labor.	Balanced nutrition and mineral supplementation, especially in late gestation; regular herd dietary assessments.
Hormonal imbalance	Hormone levels (e.g., oxytocin, prostaglandins) influence labor progression.	Reduced or excessive contractions can lead to mispositioned fetus.	Higher risk of labor dystocia, fetal hypoxia, and prolonged calving times.	Hormone therapy under veterinary supervision to manage contractions; pre-breeding hormonal assessments.
Uterine abnormalities	Structural issues like uterine torsion or adhesions can alter fetal positioning.	Increased incidence of malposition and malposture.	Compromised blood flow to fetus; high probability of cesarean section; risk of fetal hypoxia.	Veterinary assessment and, if necessary, surgical intervention; close monitoring of high-risk animals.
Fetal size	Fetal macrosomia due to genetics or maternal diet can hinder proper positioning.	High probability of malposition or failure to rotate into birthing canal.	Risk of fetopelvic disproportion; trauma to both cow and calf; increased need for surgical intervention.	Monitoring fetal growth using ultrasound; dietary control to manage excessive fetal size in late gestation.
Gestation length	Extended gestation can result in larger fetuses with higher risk of dystocia.	Increased potential for malpositions due to limited uterine space.	Increased risk of fetal mortality and birthing trauma; complications during intervention.	Early pregnancy monitoring to adjust nutrition; induction of labor under veterinary guidance if gestation is prolonged.
Fetal malpresentation	Refers to abnormal positions such as breech, transverse, or head-back.	Directly results in dystocia due to improper alignment in birth canal.	High risk of prolonged labor; potential trauma to calf and cow; likely need for extensive intervention.	Ultrasound diagnosis and repositioning techniques; manual correction during early labor if detected.
Fetal malposition	Incorrect orientation, such as deviation of head or limbs from optimal position.	Causes delayed or obstructed labor; increased need for assistance.	Potential for asphyxiation in the calf; soft tissue damage in the cow; increased mortality risk.	Skilled manual correction; early intervention; use of lubricants and appropriate repositioning tools.
Fetal malposture	Abnormal posture, including limb or neck flexion, hindering normal delivery.	Hinders smooth movement through birth canal; delays delivery.	Increased incidence of birth injuries; likelihood of assisted delivery or cesarean section.	Training personnel on recognition of malposture; manual or surgical correction as appropriate.
Environmental factors	Stressful conditions like extreme temperatures can affect labor.	Increases tension in maternal musculature, potentially disrupting positioning.	Elevated risk of preterm labor or stillbirth; maternal fatigue and reduced uterine contractility.	Controlled environment during late pregnancy; minimize handling and stress exposure for near-term animals.
Inadequate exercise	Lack of movement can reduce muscular tone, affecting labor progress.	Contributes to fetal malposition and weak uterine contractions.	Higher risk of prolonged labor; calf distress and possible hypoxia.	Encourage moderate exercise for pregnant cattle; avoid confinement during the last trimester.
Confinement and housing	Small spaces limit natural movements, leading to reduced maternal fitness.	Increased difficulty with fetal positioning due to restricted movement.	Longer labor times; fatigue in cows and potential fetal hypoxia.	Provide ample space for movement; encourage natural locomotion; housing designs that reduce confinement.
Seasonal effects	Variations in temperature and humidity may influence hormonal balance.	May result in delayed fetal positioning adjustments, complicating delivery.	Risk of labor complications, maternal distress, and inadequate fetal positioning.	Climate-controlled environments for high-risk animals; adjust breeding cycles to avoid extreme weather.
Parity effects	First-time calvers often experience higher dystocia rates due to inexperience.	Higher likelihood of improper fetal positioning and malposture.	Greater risk of fetal and maternal injury; need for increased monitoring and assistance.	Extra monitoring and support for primiparous cows; experience-based assessment for multiparous cows.
